# CORE-VNS: Dosing and titration of VNS therapy in contemporary clinical practice

**DOI:** 10.1016/j.ebr.2026.100847

**Published:** 2026-01-22

**Authors:** Ryan Verner, Francesca Beraldi, Michal Tzadok, Firas Fahoum, Riëm El Tahry, Michael A. Gelfand, George Morris, Gholam K. Motamedi, Muhammad Zafar, Arjune Sen, Massimiliano Boffini, Charles Gordon, Maxine Dibué, For the CORE-VNS Study Group

**Affiliations:** a LivaNova PLC (or a subsidiary), United Kingdom; b Faculty of Medical & Health Sciences, Tel Aviv University, Edmond and Lily Safra Children’s Hospital, Sheba Medical Center, Israel; c Faculty of Medical & Health Sciences, Tel Aviv University, Neurological Institute, Tel Aviv Sourasky Medical Center, Israel; d Institute of Neuroscience, Université Catholique de Louvain, Centre for Refractory Epilepsy, Cliniques Universitaires Saint-Luc, Belgium; e University of Pennsylvania, Perelman School of Medicine, Department of Neurology, United States; f Ascension Wisconsin, Saint Mary’s Medical Center, United States; g Georgetown University, Department of Neurology, United States; h Duke University School of Medicine, United States; i University of Oxford, Nuffield Department of Clinical Neurosciences, United States

**Keywords:** Vagus nerve stimulation, CORE-VNS, Dosing, Titration, Scheduled programming

## Abstract

In this analysis of dosing and titration in the CORE-VNS study, we aimed to replicate previously published findings related to dosing and titration of VNS Therapy and describe the impact of Scheduled Programming (SP).Participants who received their first VNS Therapy device during the CORE-VNS study and who attended at least one of either the 6- or 12-month follow-ups were selected for this analysis. Various statistical models (generalized linear mixed models, weighted Cox regression, Kaplan-Meier, and Poisson regression) were used to assess the relationship between VNS titration and clinical response. Participants who were predominantly manually titrated were compared to those who were predominantly titrated using SP. 526 participants met the inclusion criteria for this analysis. The majority were titrated manually (n = 364), compared with the SP feature (n = 162). We found a strong relationship between speed of titration and onset of clinical response but did not find SP use to significantly impact time-to-dose. The mean time-to-response in the SP group was 7.8 months, compared to a mean time-to-response of 10.7 months for manually titrated patients but this effect was not significant in the Cox regression. Patients who were titrated using SP were able to complete their titration phase with fewer required in-office visits than manually titrated patients (p < 0.0001). We replicate prior findings that titration speed of VNS impacts the time to response. Scheduled Programming does not appear to strongly impact titration speed but aids the clinical workflow and reduces patient burden by reducing the frequency of required in-office titration visits.

## Highlights

• Scheduled Programming reduces office visit burden and overall variability of titration.
• Rapid titration of VNS Therapy is associated with faster onset of clinical benefit.
• Scheduled Programming is not yet widely utilized in clinical practice.

## 1 Introduction

Drug-resistant epilepsy (DRE) is characterized by failure to respond to two appropriately selected and adequately tolerated anti-seizure medications [1]. For people with DRE, surgical and dietary approaches can help limit the severity or frequency of their seizures, often improving quality of life. Neuromodulation is growing in popularity, but clinical benefit is often not fully achieved due to a lack of clear understanding of how to program the neuromodulation devices to an effective “dose”. Nonmedication interventions for DRE must be appropriately selected and, in the case of neuromodulation, titrated to optimal effect at the patient level.

Vagus nerve stimulation (VNS) became available to patients in the United States and Europe in the mid-1990s and has since become the most widely available surgical intervention for DRE among patients who are not candidates for surgical resection or ablation. After placement of the VNS device, patients ideally undergo a 12-week titration period [2], [3]. Some patients require more time to titrate to the therapeutic dose range due to the logistics of attending a clinic or due to side effects, which are often transient or reduced in severity after habituation to stimulation. The typical target dose for VNS Therapy in contemporary practice is 1.75 mA at 250 µsec, but following the strength-duration relationship of neural stimulation can alternatively range from 1.5 mA at 500 µsec or 2.25 mA at 130 µsec [2], [4]. The target dose is based on biophysical data representing the likelihood of activating clinically relevant nerve fibers within the cervical vagus nerve [5], [6], [7]. The biophysically defined target dose of 1.75 mA at 250 µsec, is congruent with the population-level target dose – not an individually optimal dose [8]. Dosing and titration recommendations are formally described in professional society guidance [9], [10], as well as the product’s physician’s manual, however, observational evidence of practice habits suggests that these recommendations are only adopted by certain centers and clinicians [11]. A recently conducted retrospective study has suggested that VNS is most effective near the labeling-recommend target dose, especially when this dose is achieved within 3 months of implantation [8], [12].

CORE-VNS is a global, multi-center, post-market observational study and represents the most contemporary evidence for the effectiveness of VNS Therapy and its current feature set [13]. Herein, we analyze a VNS-naïve population within CORE-VNS to better understand how dosing and titration, especially during the first 12 months, impact long-term clinical outcomes in uncontrolled clinical settings, and if contemporary VNS features impact practice or outcomes. The current work focuses on the Scheduled Programming (SP) feature – an automation feature of the M1000 “SenTiva” VNS Therapy System that permits the device to change the VNS dose at a predetermined future date and time, programmed in advance by the clinician.

## 2 Methods

### 2.1 CORE-VNS study

The CORE-VNS Study is a global, post-market observational study of patients with drug-resistant epilepsy treated with the VNS Therapy System adjunctive to anti-seizure medications. The objective of the study is to evaluate real world clinical and safety outcomes of patients treated with VNS Therapy. Participants in the study were selected based on a clinical diagnosis of drug-resistant epilepsy and suitability to be treated with VNS per the clinical judgement of the site investigator. There were no exclusion criteria for this all-comers study. Participants were followed for up to 3 years with visits at 3, 6, 12, 24, and 36 months, and clinical outcome assessments collected at each visit were compared to the within-subject baseline visit data. Outcomes assessed include seizure frequency and seizure response status by seizure type as well as seizures by onset classification (≥50% reduction from baseline in seizure frequency). Other non-seizure outcomes were collected but are not the subject of the current work.

### 2.2 Population selection for dosing analysis

The CORE-VNS Study (NCT03529045) included both participants receiving their first VNS device as well as those receiving battery replacements. For the purposes of this interim analysis of dosing and titration habits, we analyzed the population who received their first VNS device as part of the CORE-VNS Study to focus on titration schedules. There were 531 participants enrolled in the CORE-VNS Study slated for initial implant as part of the study. Of those, 526 were successfully implanted and completed follow-up visits after the implant date, including at least one of the 6- or 12-month follow-up visits.

Comparator subpopulations were defined based on usage of the SP feature, which was hypothesized to be the most impactful feature on the rate of titration. Initially, we examined 2 populations: those who used the SP features and those who did not (including those who did not have access to the feature based on the implanted generator model). During analysis, we identified patterns of feature use that led to further refinement of the interim analysis population. Specifically, we further specified the subpopulation of participants who were programmed using SP as using the feature for 3 or more programming steps. This decision was made because, after examining the specific timing of SP steps, we found that a small but significant number (n = 22) of SP users only used the feature to replace 1 or 2 programming steps at different times. Participants of this “1–2 step” group were primarily manually titrated, only occasionally using SP as a replacement for an expected titration visit conflict. While this is a viable use case for SP, patients titrated using sporadic SP events more closely resembled manually titrated patients. As a result of this decision, we describe the principal outcomes for those patients who were titrated using 3 or more SP steps versus those patients who were titrated without SP.

A “responder only” sensitivity analysis was conducted, as previous literature demonstrated that our Cox Regression methodology for time-to-response may be sensitive to inclusion of patients who don’t respond to therapy.

### 2.3 Dosing and titration analysis

For this analysis that focuses on dosing and titration, we elected to examine only the 3, 6, and 12-month visits. This decision was made for two primary reasons. First, it is possible and common to complete titration of VNS Therapy and perform an initial clinical assessment of treatment effect in 12 months or less [11]. Second, existing literature suggests that faster titration of VNS results in earlier onset of clinical response [12]. As such, we hypothesized that participants titrated quickly within our study population would experience more robust clinical benefit within this 12-month window than those titrated more slowly.

Parameter selections for each subpopulation at each of the 3, 6, and 12-month study visits are provided using the most appropriate descriptive statistic for that parameter. For example, for more continuous variables such as output current, the median is provided, whereas for more categorical variables (such as frequency) the mode is provided.

Time-to-event analyses including time-to-dose or time-to-response are displayed visually using Kaplan-Meier models due to their accessibility, but hypothesis testing was conducted using Weighted Cox Regressions to better handle interaction effects. The dose target for the time-to-dose analysis was determined by achievement of the VNS Therapy System labeling-defined dosing targets for titration, namely 1.5 mA at 500µsec, 1.75 mA at 250µsec, or 2.25 mA at 130µsec. If patients met or exceeded the output currents for any of these pulse widths, they were considered to have achieved the target dose of VNS.

### 2.4 Seizure outcomes

Seizure outcomes were assessed in our models as responder status (≥50% reduction in seizure frequency from baseline) at a given time point in days post-implant. Responder status was calculated using the percentage change between the monthly average seizure frequency in the 3 months prior to each study visit, compared to the monthly average seizure frequency in the 3 months prior to baseline. For example, at the 3-month visit, the evaluation period was 0–3 months post-implant and was compared to the within-subject baseline of −3 to 0 months pre-implant. While all seizures were recorded, only those seizures present in the baseline evaluation period were used to calculate a percentage change from baseline. When reported directly, the subgroup responder rate is provided as a ratio of the participants in response at a given visit over the total number of participants reporting at that visit (missing data excluded).

### 2.5 Statistical analysis and database considerations

This analysis was conducted under a prospective statistical analysis plan collaboratively developed by authors RV, FB, MD, FF, MT, and RT. The analysis plan was ratified by the entire analysis team prior to viewing the data. Statistical programming was supervised by the study biostatistician, FB. Results of all analysis phases were reviewed by the entire analysis team. Decisions for modifications to the analysis plan were agreed by the analysis team before proceeding.

Generalized linear mixed models were used to assess the likelihood of an event. Kaplan-Meier models were used to display time-to-event analysis, with the multivariate analysis performed using weighted Cox regression. Weighted Cox regression was employed over the Cox Proportional Hazards approach due to failure of the proportional hazards assumption (specifically: time-on-therapy represented a non-proportional hazard in our model). Poisson regression was used for multivariate regressions not related to time-to-event analysis.

An analysis of responder rates at 3, 6, 12, 24, and 36 months after implantation with VNS was conducted. Comparisons between treatment groups were made using Fisher’s Exact Test.

## 3 Results

### 3.1 Population

Five-hundred thirty-one (531) participants were included in the modified safety population of the CORE-VNS study. Of the safety population, 526 participants qualified for this analysis on account of successful implantation and available follow-up data up to 12 months. In this population, the majority of participants were not programmed using SP (n = 364), including 82 participants who received a M1000 SenTiva VNS Therapy System but did not use the SP feature. Of participants who did use the feature for at least one programming visit (n = 162), most (n = 140) used the feature to program 3 or more titration steps.

There were limited differences in the clinical or demographic profiles of SP users and non-users (Table 1). Participants in the CORE-VNS study were predominantly adult epilepsy patients with highly refractory epilepsy. Participants failed a median of 6 anti-seizure medications (range 2–20) in their history of epilepsy care, which had lasted for a median of 10 years (range 2–62.5 years) before consenting into our study. Most patients experienced at least one focal seizure type at baseline and were reported to have Focal or Combined/Mixed Epilepsy (82.7%). The differences between users and non-users of SP were noted in regions of use. SP feature access was primarily available at US and European sites during the CORE-VNS study.

**Table 1:** Demographics and epilepsy history.

	Never used SP (n = 364)	Used SP 3 or more times (n = 140)	Total first implant population (n = 526)
Male Female	181 (49.7%) 183 (50.3%)	81 (57.9%) 59 (42.1%)	271 (51.5%) 255 (48.5%)
Age at First Implant (years)	21.8 1.0–71.5	19.0 2.9–71.0	20.4 1.0–71.5
Age Group at First Implant in Study	<18: 155 (42.6%) ≥18: 209 (57.4%)	<18: 66 (47.1%) ≥18: 74 (52.9%)	<18: 237 (45.1%) ≥18: 289 (54.9%)
Duration between Epilepsy Diagnosis and Informed Consent (years) †	10.0 0 – 62.5	9.75 1 – 60	10.0 0 – 62.5
Epilepsy Type	Combined: 122 (33.5%) Focal: 168 (46.2%) Generalized: 67 (18.4%) Unknown: 7 (1.9%)	Combined: 59 (42.1%) Focal: 69 (49.3%) Generalized: 11 (7.9%) Unknown: 1 (0.7%)	Combined: 190 (36.1%) Focal: 245 (46.6%) Generalized: 82 (15.6%) Unknown: 9 (1.7%)
Number of Prior ASMs Failed (#)	6.0 2–20	7.0 2–17	6.0 2–20
Generator Model*	M1000: 82 (22.5%) M106: 235 (64.6%) M103/102: 48 (13.2%)	M1000: 140 (100%) M106: 1 (0.7%)	M1000: 244 (46.4%) M106: 236 (44.9%) M103/102: 48 (9.1%)
Region US/EU Western Pacific East Med SEA	222 (61.0%) 120 (33.0%) 15 (4.1%) 7 (1.9%)	140 (100%)	384 (73.0%) 120 (22.8%) 15 (2.9%) 7 (1.3%)

*Generator models do not add to 100% because 2 subjects experienced a generator revision prior to the study’s first effectiveness evaluation.

† Duration between Age at Diagnosis and Informed Consent in Study is a derived variable of integer age. Participants with age at diagnosis <1 year were defined to have age 0.5 at diagnosis.

### 3.2 Dosing and titration

We examined the VNS settings programmed at the 3, 6, and 12 month visits using descriptive statistics for SP users and non-users (Table 2). For the median output currents and most common settings, we found very little difference between groups, especially in the longer follow-up. At the 3-month visit, the median output current of the manual titration group was higher than the median output current of participants who used any amount of SP to aid in titration. AutoStim Mode output currents and Magnet Mode output currents are typically programmed to 0.125 and 0.25 mA higher than Normal Mode output current, respectively, which appeared to be represented in our cohort. Providers tend to shift their patients to lower sensitivity settings for the AutoStim feature as they approach the target dose range (per labeling) or settle on a patient-specific target dose, but in our cohort, this change was more apparent in the SP user population and may represent a specific and uncontrolled confound in our analysis. Critically, the move to lower thresholds for Autostim in the SP group would have the effect of increasing the overall duty cycle of VNS, as lower Autostim thresholds are associated with a higher number of Autostimulation events per day. Furthermore, patients in the SP group were more likely to use lower signal frequencies compared to the manual titration group. This is likely an effect of the default settings in the SP feature (defaults to 20 Hz setting, as opposed to 30 Hz). While this is a notable difference, recently literature has questioned the significance of the impact of signal frequency selection (between 20 and 30 Hz) on clinical outcomes [8].

**Table 2:** VNS parameters at the 3, 6, and 12 month visits.

Never Used SP	3mo	6mo	12mo
Normal Mode Median OC (range) Mode PW (%) Mode SF (%) Mode DC On (%) Mode DC Off (%)	1 mA (0–2.25 mA) 250µsec (50.5%) 30 Hz (46.7%) 30 sec (70.3%) 5 min (60.2%)	1.375 mA (0–2.5 mA) 250µsec (52.7%) 30 Hz (41.8%) 30 sec (64.0%) 5 min (46.2%)	1.750 mA (0–3.0 mA) 250µsec (62.1%) 30 Hz (42.9%) 30 sec (69.5%) 5 min (38.7%)
AutoStim Mode Median OC (range) Mode PW (%) Mode On Time (%) Threshold (%)	1.0 mA (0–2.25 mA) 250µsec (40.4%) 30 sec (37.1%) 40% (28.8%)	1.375 mA (0–2.75 mA) 250µsec (40.7%) 30 sec (36.8%) 40% (23.4%)	1.750 mA (0–3.25 mA) 250µsec (44.5%) 30 sec (39.3%) 40% (24.2%)
Magnet Mode Median OC (range) Mode PW (%) Mode On Time (%)	1.25 mA (0–2.5 mA) 500 µsec (42.6%) 60 sec (64.3%)	1.625 mA (0–3.0 mA) 500 µsec (38.5%) 60 sec (59.6%)	2 mA (0–3.25 mA) 500 µsec (42.3%) 60 sec (67.6%)

Used SP 3+	3mo	6mo	12mo
Normal Mode Median OC (range) Mode PW (%) Mode SF (%) Mode DC On (%) Mode DC Off (%)	0.5 mA (0–2.0 mA) 250µsec (85.0%) 20Hz (51.4%) 30 sec (83.6%) 5 min (82.9%)	1.5 mA (0–2.5 mA) 250µsec (80.7%) ≤20Hz* (49.3%) 30 sec (82.1%) 5 min (66.4%)	1.75 mA (0–2.5 mA) 250µsec (80.7%) ≤20Hz* (48.6%) 30 sec (75.0%) 5 min (40.0%)
AutoStim Mode Median OC (range) Mode PW (%) Mode On Time (%) Threshold (%)	0.625mA (0–2.25 mA) 250µsec (85.7%) 60 sec (60.7%) 40% (35.0%)	1.625mA (0–2.5 mA) 250µsec (81.4%) 60 sec (54.3%) 30% (28.6%)	1.875mA (0–2.75 mA) 250µsec (81.4%) 60 sec (49.3%) 20% (35.7%)
Magnet Mode Median OC (range) Mode PW (%) Mode On Time (%)	0.75 mA (0–2.5 mA) 500 µsec (70.7%) 60 sec (82.1%)	1.75 mA (0–2.75 mA) 500 µsec (63.6%) 60 sec (80.0%)	2.0 mA (0–2.75 mA) 500 µsec (60.7%) 60 sec (78.6%)

* at the 6 month visit, only 1 participant was programmed to a Signal Frequency <20 Hz. At the 12 month visit, only 2 participants were programmed to a Signal Frequency <20 Hz.

### 3.3 Time-to-dose analysis

**Fig. 1:**
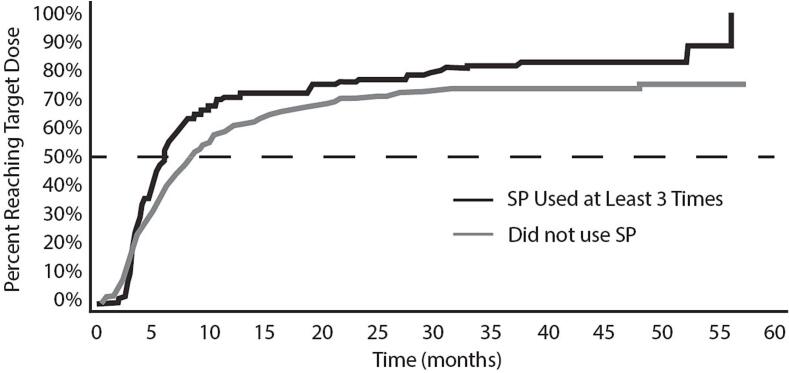
Time (months) required by participants in the CORE-VNS study to achieve the target dose range per the VNS Therapy Physicians Manual.

In our assessment of time-to-dose (Fig. 1), we compared participants titrated using primarily SP (at least 3 SP events) versus those who were titrated using manual titration. Kaplan-Meier analysis demonstrates a numerically distinct impact of SP use on time-to-dose (Fig. 1), and this effect seems to be driven by variance and especially a longer tail to the time-to-dose distribution within the manually titrated group. The mean time-to-target-dose was 4.9 months (median 4.1 months, range 0–11 months) when SP used. In contrast, manually titrated participants achieved the target dose with a mean time of 5.1 months (median 4.6 months, range 0–12 months). This difference was not significant (Cox Regression; p = 0.10).

### 3.4 Time-to-response analysis

**Fig. 2:**
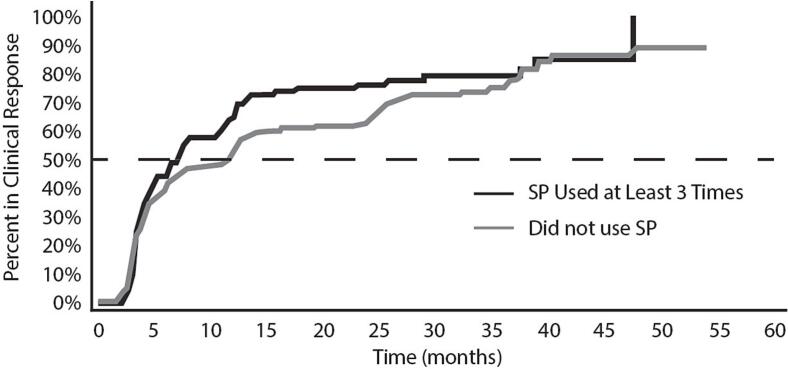
Time (months) required by participants in the CORE-VNS study to achieve clinical response (50% reduction in seizure frequency compared to baseline).

In our assessment of time-to-response (Fig. 2), we compared participants titrated using primarily SP (at least 3 SP events) versus those who were titrated using manual titration. As with time-to-dose, the Kaplan-Meier analysis visually suggests a relationship between SP use and time-to-response that is ultimately not significant. The mean time-to-response in the SP group was 7.8 months (median 4.5; range 2.1–47.6), compared to a mean time-to-response of 10.7 months (median 5.6, range 1.5–47.6). This effect was not significant when assessed in a multivariate Cox Regression. In fact, Cox Regression revealed a non-significant inverted effect of SP on time-to-response – suggesting that use of SP may delay response.

At the same time, our model identified a strong and statistically significant (p < 0.05) correlation between time-to-dose and time-to-response, replicating findings of Tzadok et al 2022. Further investigation revealed our Cox model to be highly sensitive to inclusion of participants who never responded to VNS. When only participants who eventually responded to VNS were included in the model (both SP users and non-users), positive and negative correlations were strengthened. The restricted model indicates SP use was associated with longer time-to-response (p < 0.001) while faster titration was strongly associated with shorter time-to-response (p < 0.001).

### 3.5 Impact of SP on clinical outcomes

Use of Scheduled Programming was not independently associated with superior clinical outcomes (Table 3, Table 4). After 12 months of VNS, those patients titrated with the help of Scheduled Programming experienced a median 53.5% reduction in seizure frequency for all seizures, compared to 52.4% reduction for those participants who were manually titrated. Responder rates were similar between groups as well, with 56.2% of SP participants responding to therapy at 12 months compared to 53.6% of manually titrated participants.

**Table 3:** Responder rate at each follow-up.

	Statistics	3 Months	6 Months	12 Months	24 Months	36 Months
Used SP at least 3 times	N	120	122	121	116	110
	n (%)	56 (46.7%)	66 (54.1%)	68 (56.2%)	66 (56.9%)	68 (61.8%)
	95% CI	38.9% to 54.6%	46.3% to 61.8%	48.3% to 63.9%	48.8% to 64.7%	53.6% to 69.6%
Never Used Scheduled Programming	N	265	263	293	278	269
	n (%)	127 (47.9%)	121 (46.0%)	157 (53.6%)	177 (63.7%)	178 (66.2%)
	95% CI	42.7% to 53.2%	40.8% to 51.3%	48.6% to 58.5%	58.7% to 68.5%	61.1% to 71.0%
	P-value	0.83	0.15	0.67	0.21	0.48

**Table 4:** Percentage change in seizure frequency (compared to baseline) at each follow-up.

	Statistics	3 Months	6 Months	12 Months	24 Months	36 Months
Used SP at least 3 times	N	120	122	121	116	110
	Median Percentage Change	−40.0%	−50.8%	−53.5%	−58.8%	−76.7%
	95% CI	−55.6% to −25.0%	−68.2% to –33.3%	−66.7% to –33.3%	−85.0% to −40.0%	−89.7% to −53.3%
Never Used Scheduled Programming	N	265	263	293	278	269
	Median Percentage Change	−44.4%	−43.3%	−52.4%	−68.3%	−77.5%
	95% CI	−50.0% to −33.3%	−52.2% to −28.6%	−66.7% to −42.9%	−77.8% to −60.2%	−86.2% to −66.7%
	P-value	0.95	0.10	0.78	0.39	0.70

For participants who were manually titrated (never used SP), the mean number of titration visits required to achieve the target dose range per the VNS Therapy System labeling was 5.44 visits (median 6, range 1–13). Using SP to support titration (3 or more steps) reduced the mean number of visits to 3.3 (median 3, range 1–10), a significant reduction (Poisson estimate −0.53; p = <0.0001).

### 3.6 Adverse events

Adverse events in the study population more commonly reported in manually titrated patients (n = 151/364; 41.5%) than in patients using SP 3 or more times (40/140; 28.6%). Adverse events were predominantly stimulation-associated (rather than procedure-associated) and were dominated by dysphonia (17.3% of manual, 10.7% of SP), dyspnea (8.0% of manual, 4.3% of SP), and cough (6.9% of manual, 4.3% of SP). Adverse events were typical of general clinical experience with VNS Therapy.

## 4 Discussion

Our study indicates that SP supports the titration of patients receiving VNS for DRE by automating the titration process outside of regularly scheduled office visits. Participants in the CORE-VNS study who were primarily titrated using SP (≥3 titration events using SP) were significantly more likely to achieve the labeling-defined target dose with fewer in-office titration visits. This is of key importance to families with epilepsy who bear the burden of such visits: planning, taking time from work, travel, risk to the patient that travel might cause, anxiety to the family, etc. It is of additional value to the office staff, who must allocate valuable staff time to program the device the next step in a dosing regimen. Furthermore, time-to-dose was more consistent (less variance in time-to-dose) for patients titrated using SP.

In clinical practice, dosing is mainly dependent on 3 factors: tolerability of higher intensity settings, ability of the patient to attend regular titration visits (including limitations on clinic availability), and institutional or practitioner titration habits or protocols that inform the process. We argue that SP supports the titration process via all three of these factors. First, while large changes in VNS intensity are known to evoke tolerability complaints in patients, smaller intensity increases have been shown to result in steady adaptation or habituation to increased intensity [3]. SP’s standard protocol leverages a biweekly 0.25 mA current increase that has been shown in randomized controlled trials to manage tolerability concerns while still moving patients on the path to therapeutic VNS doses. Second, SP decreases the need for patients to attend in-office titration visits – with SP, office visits are needed only in case of a tolerability issue. In our study, any use of SP to support the titration process resulted in significant reduction in office visits required for titration, and the reduction was greater for those who used SP for more titration steps. The fact that we observed fewer office visits during the titration phase for the SP group supports the hypothesis that unplanned visits to address tolerability issues related to SP are still less frequent than repeated planned office-visits for traditional titration. Finally, SP provides a common standard protocol for titration that all providers can follow, removing some of the uncertainty and difficulty in programming VNS. The protocol is evidence-based, aligned to professional guidance, and is proven to reliably deliver patients to the target dose range with manageable tolerability [3], [9].

Titration guidance for neurostimulation therapies is fundamentally different than that of pharmaceutical therapies because electrical neurostimulation operates on bioelectric principles while pharmaceuticals are often dependent on pharmacokinetic and pharmacodynamic properties. Principally, the difference in therapeutic titration of VNS versus medications relies on the all-or-none nature of neuronal activation (and subsequent summation of many individual potentials into a compound action potential). Pharmaceutical therapeutics operate on cellular mechanisms that modulate internal, external, or transmembrane protein activity or expression, neurostimulation modalities operate directly on voltage-gated transmembrane proteins responsible for the action potential. Because voltage-gated mechanisms are all-or-none, electrical stimulation therapies like VNS require that a patient-specific charge density be achieved before that patient can benefit from therapeutic stimulation (though the requirement for sufficient nerve bundle activation can lead to some variance in patient-level dosing requirements).

The comparatively small population using SP (n = 162 using any SP versus n = 364 using none) in the CORE-VNS study suggests that SP is not yet routinely employed in clinical practice. This is in line with results from a recently published global survey of VNS users indicating that only approximately a third of VNS users utilize SP to titrate patients [11]. One important consideration for this finding is that CORE-VNS is a global registry, and not all regions participating in the study have access to the SenTiva model (the only VNS model that includes SP), or they gained access to the model later during the enrollment window and were less familiar with the technology. In CORE-VNS, approximately 1 in 3 patients had their dose of VNS Therapy titrated with the SP feature, and 1 in 3 SenTiva users are not titrated using SP in any capacity. Demographic factors that might influence the likelihood of using the feature, such as the age or cognitive status of the patient, do not appear to be major drivers of utilization (though children were numerically more likely to be programmed with SP). We hypothesize that access to the feature and confidence in managing tolerability concerns outside of the clinic are likely two primary drivers of low utilization of SP. However, with the data presented here, providers can begin to reconsider this approach, weighing the access benefits (reduced in-office visit burden – for the patients and clinic staff) identified in our study against the risk of transient tolerability issues outside of the clinic.

It is important to recognize that manual titration can follow the same schedule as SP, and some clinicians titrate even faster than the SP standard titration protocol. There were manually titrated participants in CORE-VNS who achieved the target dose range as quickly as or more quickly than participants titrated using SP, which was likely a confounder in our time-to-response Cox Regressions. One important replicated finding in our study was that time-to-dose was strongly and significantly correlated with time-to-response, as has now been described in two independent cohorts. [12], [14] Our results clearly indicate that use of SP is not a requirement to achieve faster response and may actually impede response if patients can tolerate faster titration speeds than SP allows. A balance between in-office visits to maximize titration while maintaining tolerability, supplemented with out-of-office SP events to continue to advance titration steps, may be the most pragmatic approach to titration in the absence of supportive telemedicine technologies. Ultimately, the objective is faster titration – as faster titration has been repeatedly shown to yield faster response. [12], [14].

Our study has many limitations. Time-to-dose and time-to-response can both be impacted by multiple clinical factors excluding the impact of SP. Time-to-dose can be impacted by clinical factors like the presence of adverse events or side effects related to therapy, or non-clinical factors like the distance between the patient’s home and their clinician’s office. Time-to-response can be impacted by patient-level factors that influence the overall likelihood to respond, or to respond at lower overall VNS intensities that can be achieved earlier in the titration process. Rigorous matching (e.g. propensity score matching) of the Scheduled Programming vs Manual Titration cohorts based on demographic or clinical variables was not performed, and as the study was observational, controls for dosing behaviors such as controlling signal frequencies, autostimulation use, and duty cycles was not performed. Instead, controls for covariates were handled in our statistical approach. Assessment of these demographic or physiological variables is unfortunately outside the scope of this report. Our study was not designed to control for these factors, nor collect necessary data to investigate them thoroughly. While CORE-VNS is a large study, the overall number of frequent SP users was lower than anticipated. Access to SenTiva systems certainly contributed to the higher number of participants who did not undergo SP because the SenTiva pulse generator was not commercially available in all study regions at the time of study initiation.

## 5 Conclusion

SP can improve the consistent and expedient titration of VNS, but ultimately the objective of rapid onset of seizure reduction is driven by rapid titration – with or without SP. While our evidence supports use of SP holistically to reduce overall burden to patients and clinicians, when SP is not used or not accessible, effort should be made to achieve the target dose range as quickly as possible, as this practice is associated with superior clinical outcomes.

## 6 Ethical publication statement

All authors affirm their acknowledgement and commitment to the ethical publication principles maintained by the journal, including that this manuscript is representative of original research that was conceptualized, drafted, and approved for publication by all authors.

## CRediT authorship contribution statement

**Ryan Verner:** Writing – original draft, Project administration, Methodology, Conceptualization. **Francesca Beraldi:** Writing – original draft, Methodology, Formal analysis. **Michal Tzadok:** Writing – review & editing, Investigation, Conceptualization. **Firas Fahoum:** Writing – review & editing, Investigation, Conceptualization. **Riëm El Tahry:** Writing – review & editing, Investigation, Conceptualization. **Michael A. Gelfand:** Writing – review & editing, Investigation. **George Morris:** Writing – review & editing, Investigation. **Gholam K. Motamedi:** Writing – review & editing, Investigation. **Muhammad Zafar:** Writing – review & editing, Investigation. **Arjune Sen:** Writing – review & editing, Supervision, Investigation. **Massimiliano Boffini:** Writing – review & editing, Methodology, Conceptualization. **Charles Gordon:** Writing – review & editing, Supervision, Methodology, Formal analysis, Conceptualization. **Maxine Dibué:** Writing – review & editing, Supervision, Conceptualization.

## Declaration of competing interest

The authors declare the following financial interests/personal relationships which may be considered as potential competing interests: RV, FB, MB, CG, and MD are employees of LivaNova PLC (or a subsidiary), the manufacturer of the VNS Therapy System, and have received salary compensation as well as hold stock or stock options. MT, FF, RET, MAG, GM, GKM, MZ, and AS were CORE-VNS study investigators, and they or their investigational sites have received financial support from LivaNova PLC (or a subsidiary) as part of their role in the CORE-VNS study in order to conduct the study. While time spent performing study related activities may have been compensated according to investigational site agreements, no compensation was offered with respect to the development of this manuscript. The authors report no patents or intellectual property related to this work.
