# Supplementary material for: CORE-VNS: Dosing and titration of VNS therapy in contemporary clinical practice
Source: Epilepsy Behav Rep. 2026 Jan 22;33:100847. doi: 10.1016/j.ebr.2026.100847 (PMC12950443; doi:10.1016/j.ebr.2026.100847)
Supplement: Supplementary Data 1 [file mmc1.pdf]

AUSTRALIA	Site Contacts
Queensland Children’s hospital	Sophie Calvert
	Kate Riney
The Royal Children’s Hospital	Jillian Bicknell-Royle
	Jermey Freeman
	Simon Harvey
	Kathryn Santamaria
The Royal Melbourne Hospital	Hue Mun Au Yong
	Lei Chen
	Karen Chidgey
	Amy Ellis
	Chris French
	Darren Germaine
	Ofer Gonen
	Zoe Holding
	Sean Hosking
	Elle Hurley
	Patrick Kwan
	Samantha Mackwell
	John-Paul Nicolo
	Andrew Pattichis
	Piero Perucca
	James Ryan
	Lubna Shakhatreh
	Marian Todaro
	Anna Willard
The Alfred Hospital	Katherine Burn
	Stephanie Chamorro
	Emma Foster
	Emily Galea
	Jack Germaine
	Rachel Hines
	Mubeen Janmohamed
	Patrick Kwan
	Joshua Laing
	Andrew Neal
	Terence O'Brien
	Alison Ottrey
	Andreas Pattichis
	Alex Philippou
	Georgia Ramsay
	Lubna Shakhatreh
	Rafael Smith
	Lily Turner
Perth Children's Hospital	Nicole Miglori
	Lakshmi Nagarajan
	Snehal Shah
	Jessie White

AUSTRIA	
Kepler Universitätsklinikum Neuromed Campus	Elke Bach
	Astrid Eisenkolbl
	Gudrun Groeppel
	Martin Hamberger
	Anna Maria Hengsberger
	Manuel Puhringer
	Romana Schiller
	Gabriele Schwarz
	Tim J. von Oertzen
	Lisa Ameshofer
	Gertraud Puttinger
	Sonja Thalguter
**BELGIUM**	
UCL - Cliniques universitaires Saint-Luc	Venethia Danthine
	Manon Dumoulin
	Riem El Tahry
	Susana Ferrao Santos
	Simone Vespa
	Pascal Vrielynck
UZ Gent	Louise Adams
	Helen Bachmann
	Arnout Bruggeman
	Sofie Carrette
	Laura Couvreur
	Veerle De Herdt
	Elien De Schampehelaere
	Louise De Temmerman
	Zoë Delaruelle
	Camille Dutordoir
	Stefanie Gadeyne
	Ieme Garrez
	Sebastien Heyndrickx
	Stephanie Höedl
	Helena Janssen
	Tim Kelderman
	Ann-Sophie Lamon
	Simon Lamquet
	Elien Lecomte
	Griet Loret
	Ann Mertens
	Antoon Meylemans
	Heleen Parmentier
	Mathieu Sprengers
	Jonas Toeback
	Sofie Van Assche
	Annelore Van Bleyenbergh
	Daan Van Den Abbeele

	Astrid Van Den Broecke
	Julie Van Houtte
	Vincent Van Iseghem
	Kato Van Rooy
	Alexander Vanhoorne
	Soetkin Vantyghem
	Marijke Vergaelen
	Kristl Vonck
**BRAZIL**	
Fundação Felice Rosso, Hospital Felício Rocho	Maria Jose Alves Tostes
	Joao Vitor Broglio De Oliveira
	Luisa Carim
	Ana Paula Gonçalves
	Andréa Julião de Oliveira
	Cristyelle Kenia Valadares Martins Linhares
	Aline Pedroso Dos Santos
	Drielly Kaizer Ramos Rocha
	Barbara Silva
	Lahis Souza De Araujo
Instituto Estadual do Cérebro Paulo Niemeyer (IEC)	Isabella D'Andrea Meira
	Nelma Veronica Marques
	Marilia Martins
	Henrique Prado
**CANADA**	
Centre Hospitalier de l'Université de Montréal	Veronique Cloutier
	Mark Keezer
	Dang-Khoa Nguyen
Montreal Neurological Institute and Hospital	Martin Chevrier
	Rick Sanchez
	Julian Santorelli
	Martin Veilleux
McGill University Health Centre, Montreal Children's Hospital	Frederique Arnaud
	Natallia Barysevich
	Anita Hsieh
	Ayan Ibrahim
	Hasmig Kavoukian
	Kenneth Myers
	Kimberly Pandolfini
	Katarina Perreault
London Health Sciences Centre	Suzan Brown
	Jorge Burneo
	Ana Suller Marti
London Victoria Hospital Pediatric Center	Andrea Andrade
	Melissa F Chavez-Castillo
	Michelle Gratton
	Dena Haile
	Rhiannon Hicks
	Jaehee Kim

	Maryam Nouri
	Rochelle Sorzano
	Daniela Yepes
**FINLAND**	
Tampere University Hospital	Peltola, Jukka
**CHINA**	
Sanbo Brain Hospital	Yuan Ding
	Qing Gao
	Yuguang Guan
	Tianfu Li
	Fei Liu
	Dongshuang Lu
	GuoMing Luan
	Junhong Pan
	Lan lan Qiu
	Xiongfei Wang
	Douyu Wen
	Jian Zhou
Renji Hospital Shanghai Jiaotong University School of [illegible]	Xinyuan Huo
	Qiangqiang Liu
	Junfeng Ma
	Changquan Wang
	JiWen Xu
	Xiaolai Ye
	Hongyu Zhou
Xuanwu Hospital Capital Medical University	Ying Guo
	Yang Hao
	Yongsheng Hu
	Xuefeng Jia
	Yongjie Li
	Jianyu Li
	Rong Li
	Mengxian Liang
	Baicheng Liu
	Mei Lu
	Kai Ma
	Liang Qiao
	Xueyuan Wang
	Yunpeng Wang
	Xinying Wu
	Shiqiang Xi
	Cuiping Xu
	Xiaoming Yan
	Tao Yu
	Xi Zhang
	Hongwei Zhu
	Jin Zhu
Guangzhou Women and Children's Medical Center	Yufeng Feng

	Fangcheng Li<br>Jingrong Lin<br>Yuting Wu<br>Xinke Xu<br>Qinglin Zhao
**INDIA**	
St. John's Medical College Hospital	Aman M. Anandan<br>Thomas Mathew<br>Raghunandan Nadig<br>Gosala Raja Sarma
P. D. Hinduja Hospital and Medical Research Centre	Prajakta Ghatage<br>Amey Kamble<br>Snehal Kelaskar<br>Neha P More<br>Himanshi Nag<br>Arati Patil<br>Abhishek P PATWARDHAN<br>Vrajesh Udani
Deenanath Mangeshkar Hospital and Research Centre	Nilesh Kurwale<br>Sandip Patil<br>Deepali Patil
**ISRAEL**	
Sheba Medical Center	Ester Frank<br>Michal Tzadok<br>Gila Zwas
ITel Aviv Sourasky Medical Center	Thaera Arafat<br>Tal Bar Adon<br>Eden Ben Yaakov<br>Olga Brusilovsky<br>Tamar Eviatar-Ribak<br>Firas Fahoum<br>Lilach Goldstein<br>Itai Loushy<br>Noa D Sheikin<br>Elena Skripai<br>Stav Wolf
Schneider Children's Medical Center of Israel	Sapirit Argaman<br>Yaara Daud<br>Hadassa Goldberg-Stern<br>Dror Kraus<br>Noam Noam Frenkel<br>Maayan Rosen
**ITALY**	
Ospedale Bellaria	Lidia Di Vito<br>Cristiano Fanciulli<br>Barbara Mostacci<br>Paolo Tinuper

Bambino Gesù Pediatric Hospital	Costanza Calabrese<br>Giusy Carfi Pavia<br>Rossana Cocchiola<br>Alessandro De Benedictis<br>Luca De Palma<br>Alessandro Ferretti<br>Cristina Filosomi<br>Chiara Pepi<br>Nicola Pietrafusa<br>Nicola Specchio
**JAPAN**	
NCNP National Center Hospital of Neurology and Psychiatry	Keiya Iijima<br>Akihiko Ishiyama<br>Masaki Iwasaki<br>Kyoko Kanazawa<br>Yuu Kaneko<br>Yukiko Kimura<br>Eiji Nakagawa<br>Takashi Saito<br>Naotake Shoji<br>Noriko Sumitomo<br>Yutaro Takayama
The University of ToKyo Hospital	Naoto Kunii<br>Seijiro Shimada<br>Kazuhiko Takabatake<br>Megumi Takasago<br>Kei Yanai
National Hospital Organization Nagasaki Medical Center	Yuko Fujioka<br>Ryoko Honda<br>Risa Kimura<br>Tomonori Ono<br>Ohki Saito<br>Yoshiaki Watanabe
**NETHERLANDS**	
Stichting Epilepsie Instellingen Nederland (SEIN)	Jansje Jacqueline Ardesch<br>Inge Brummelhuis<br>Anita Geertsema<br>Janita Glastra-Zwiers<br>Boudewijn Gunning<br>Janic Hulst - van Zijl<br>Claudia Kamsma<br>Jessica Mijnheer<br>Jesca Otterman<br>Jelisa van Ooijen - Kollen
Kempenhaeghe	A.J. (Albert) Colon<br>Ineke Diderich

	Marion Driessen-Willems
	Anneke Rampen
	Marion Savelkoul
	Monique Smulders
	Janny Van Der Steen
	Louis Wagner
Sophia Kinderziekenuis	Nienke Borst-Wezemer
	M.C.Y De Wit
	Suzanne Koudijs
	Rinze F. Neuteboom
**POLAND**	
Uniwersyteckie Centrum Kliniczne im. Prof. K. Gibińskiego	Ewa Krzystanek
	Barbara Kściuk
	Joanna Siuda
	Justyna Tabaka-Pradela
Children's Memorial Health Institute	Katarzyna Kotulska-Jozwiak
	Dariusz Kuczyński
	Agata Ulatowska
**PORTUGAL**	
Centro Hospitalar de São João	Clara Chamadoira
	Pedro Monteiro
	Sandra Pereira
	Ricardo Rego
	Helena Rocha
**SAUDI ARABIA**	
King Faisal Specialist Hospital & Research Center – Jeddah	Maiad Faez Albalawi
	Halah Alenizi
	Hussam Alhaidari
	Afnan Alkhotani
	Khaled AlQadi
	Youssef Al-Said
	Fawzi Babtain
	Amani Badokhon
	Saleh Baeesa
	Mohammed Binmahfoodh
	Ahmad Elmardenly
	Maysa Hegazi
	Osama Muthaffar
	Abdallah Tamim
King Faisal Specialist Hospital & Research Center - Riyadh	Tariq Abalkhail
	Amal Abujaber
	Hesham Al Dhalaan
	Dhefaf Alabdaly
	Alwanah Albuhairi
	Mashael Omar Alkhateeb
	Faisal Alotaibi
	Ibrahim Althubaiti
	Salah M Baz
**SPAIN**	
ESPBIO011 - Hospital Universitario Cruces	Inigo Garamendi
**UNITED KINGDOM/ENGLAND**	
Southmead Hospital (North Bristol NHS Trust)	Howard Faulkner
	Amelia Gregory
	Helen Hodgson
	Madhu Ramamoorthi
	Kasia Sieradzan
	Samantha Travis
	Geraldine Watson
King's College Hospital	Adedamola (Dammy) Adebayo
	Ivan Caro
	Sanisha Lakhani
	Hannah Martin
	David McCormick
	Debbie Miller
	Eniola Nsirim
	Benedetta Pantaloni
	Cathy Queally
	Michelle Tran
	Katie Tupper
	Antonio Valentin
Royal Victoria Infirmary Hospital	Verity Calder
	Sarah Cook
	Donald Craig
	John Davis
	Louise Dimmick
	Shan Ellawela
	Sarah Francis
	Joanne Glenton
	Danielle Hall
	Jennifer Haworth
	Ann Hudson
	Tom Jarvis
	Paul MC Alinden
	Leeanne Ratcliffe
	Donna Robson
	Lisa Robson
	Donna Ross
	Rhys Thomas
Norfolk&Norwich University NHS FT	Juliet Bransgrove
	Jeffrey Cochius
	Rebecca Cusack
	Deshanthi Elleray

	Ekkehart Staufenberg
John Radcliffe Hospital	Jane Adcock
	Paola Costa
	Charles Coston
	Ishmeet Kaur
	George Kitchen
	Amani Krayem
	Alison Mollett
	Marcus Neale
	Charlotte Parkes
	Rohini Rattihalli
	Arjune Sen
	Maggie Tristram
	Sarsha Wilson
	Charlotte Woodward
Royal Hallamshire Hospital	Ryan Asher
	Alice Brockington
	Rose Clegg
	Dena Dorniani
	Patrick Easton
	Alice Howell
	Alex Radford
	Markus Reuber
	Jacquiline Smith
**UNITED STATES**	
Child Neurology Consultants of Austin	Donovan Calvert
	Aaron Cardon
	Talitha Durgin
	Victoria Henderson
	Karen Keough
	Kate Labiner
	Brenda Rincon
	Leyra Santiago
	Offer Shavit
	Amber Steele
Dent Neurological Institute	Traci Aladeen
	Shelby Belair
	Thao Dang
	Allison Emborsky
	Marc Frost
	Jessica Greger
	Amanda Jamison
	Tatiana Jimenez-Knight
	Megan King
	Caroline Kumm
	Elinor Markowski

	Sandra Giannantonio
	Anna Mattle
	Mohammed Qasaymeh
	Michelle Rainka
	Julia Stoerr
	Rachael Wojcik
St. Mary's Hospital & Medical Center	Lisa Bertrand
	Teri Britt
	Marie Collier
	Zoe Dancy
	Eleanor Diamse
	Heidi Kaiser
	Seth A Kareus
	Michele Salaz
	Michael Yagi
Hawaii Pacific Neuroscience	Particia Borman
	Ashlea Chandler
	Catherine Chao (Mitchell)
	Samuel Kim
	Chevis Levi
	Kore K Liow
	Sheila Mahinay
	Alec Sheppard
	Jason Viereck
	Alexandra N Yankoglu
	Courtnie Yokono
	Ena Zhu
Via Christi Health	Ricky Lee
	Kelli Rice
	Toni Sadler
Wake Forest University	Jane Boggs
	Jessica Bussey
	Kelly Conner
	Daniel Couture
	Alexis Goodson
	Carolyn Hedrick
	Heidi Munger Clary
	Deanna Oates
	Cormac O'Donovan
	Gautam Popli
	Maria Sam
	Mary Silvia
	Jean Ann Trull
	Mandy Tuttle
	Valerie Woodard
Research Institute of Orlando	Kristen Batson

	Michele Cabrera<br>Michelle Clevenger<br>Revathi Ivengar<br>Miquel Jimenez<br>Heng C Lai<br>Michael Maury<br>Dhaneshwar "D" Oomrow<br>Ahmed Sadek<br>Scott Scheuermann
Le Bonheur Children's Hospital	Karen Butler<br>Stephen Fulton<br>Amy McGregor<br>Basanagoud Mudigoudar<br>Amy Patterson<br>Tracee Ridley-Pryor<br>Marianna S Rivas Coppola<br>Sarah Weatherspoon<br>James Wheless
Columbia St. Marys	Jesica Gill<br>Cynthia Lessila<br>George Morris<br>Kevin Morrow<br>Erika Olmos<br>Rebecka Snyder<br>Letrane Spears<br>Paul Subarna<br>Laura Tonyan<br>Margaret Wilson
Tulane University	Kelsey Confreda<br>Stacy Drury<br>Lauren McLester-Davis<br>Stephen L Nelson<br>Leslie Smitley<br>Brittani Wild
University of Pennsylvania	Danielle Becker<br>Kathryn Davis<br>Caitlin Davis<br>Michael Gelfand<br>Margaux Giardino<br>Sabrina Herzberg<br>Ashlie Jefferson<br>Tara Jennings<br>Melissa Johnston-Esparza<br>Emily Killian<br>Hailey King<br>Creson Lee

	Meryl Lozano<br>Alexandria Pickett<br>Kaitlyn Schott<br>Jiyoon Song<br>Bethany Thomas<br>Olivia Valazza
University of Pittsburgh Medical Center	Sandra Alhaj<br>Anto Bagic<br>Joanna Fong<br>Sherri Mosovsky<br>Dane Prince
Allegheny Neurological Associates	Patricia Blenk<br>Mike Bucklaw<br>Laurie Dennis<br>Mary Fetter<br>Kevin Kelly<br>Carole Lane<br>Miranda Nadeo<br>Devin Nelson<br>Jason Sumner<br>James Valeriano<br>Pamela M White
Duke University School of Medicine	Brisa Barajas-Gomez<br>Melissa Mc Lean<br>Hannah Nations<br>Gloria Pinero<br>Lyndsey Prange<br>Dmitry Tchapyjnikov<br>Stephanie Thera<br>Nicole Willis<br>Muhammad Zafar
University of Texas Health Science Center – San Antonio	Floyd Jones<br>Yogeet Kaur<br>Linda Leary<br>Samantha Lewis<br>Octavian Lie<br>Wesley Lowell<br>Sarah Martinez<br>Samiya Rashid<br>Charlotte Rhodes<br>Florence Wall
State University of New York	Robert Beach<br>Marielle De Masi-Posmik<br>Lena Deb<br>Muhammad Iqbal<br>Jacob Pusey

	Gaddum Reddy<br>Xiangping Zhou
Pediatric Epilepsy and Neurology Specialists	Marie D Akers<br>Jose A Ferreira<br>Mayra Ferreira<br>Kristin Fritch<br>Grace Mora<br>Susan Ngo
Georgetown University Medical Center	Alexis Ahmad<br>Silvia Bartlett<br>Luke Lovelace<br>Gholam Motamedi<br>Sandeep Pamula<br>Zsofia Parragh<br>Emma Waldon
Valley Health System	Andrea Corcoran<br>Robert Fox<br>Anne M Ganse<br>J.Craig Henry<br>Paul D. Lyons<br>Jennifer Stanford<br>Esther Tang
Albany Medical Center	Lynch, Timothy
Onsite Clinical Solutions LLC	Nahouraii, Robert
West Virginia University	Sheikh, Zubeda
Tulane University	Nelson, Stephen

The list includes those who were designated as facilitators and members of the CORE-VNS Study teams at individual sites. Some sites may have participants who are included in enrolled population but not in the mENR or MSAF populations. Some enrolled were classified as screen failures and did not receive a VNS Therapy implant.

Some participants also were found not to receive a VNS Therapy Implant due to pandemic restrictions and timelines for surgery.
